# Safety of full bowel preparation and colonoscopy in elderly patients with ulcerative colitis: A real‐world multicenter retrospective cohort study

**DOI:** 10.1002/deo2.275

**Published:** 2023-07-23

**Authors:** Yu Hashimoto, Shiko Kuribayashi, Yuki Itoi, Keigo Satou, Kou Nakata, Kengo Kasuga, Hirohito Tanaka, Hiroko Hosaka, Takashige Masuo, Kyoko Maruhashi, Kensuke Furuya, Tomoyuki Masuda, Kazuhiro Takahashi, Setsuo Yamazaki, Atsuo Iwamoto, Toshio Uraoka

**Affiliations:** ^1^ Department of Gastroenterology and Hepatology Gunma University Graduate School of Medicine Gunma Japan; ^2^ Department of Gastroenterology Isesaki Municipal Hospital Gunma Japan; ^3^ Department of Gastroenterology and Hepatology Kusunoki Hospital Gunma Japan; ^4^ Department of Gastroenterology National Hospital Organization Shibukawa Medical Center Gunma Japan; ^5^ Department of Gastroenterology National Hospital Organization Takasaki General Medical Center Gunma Japan; ^6^ Department of Gastroenterology Japanese Red Cross Haramachi Hospital Gunma Japan; ^7^ Department of Gastroenterology and Hepatology Japanese Red Cross Maebashi Hospital Gunma Japan; ^8^ Department of Gastroenterology Public Tomioka General Hospital Gunma Japan

**Keywords:** colonoscopy, elderly patients, Mayo Endoscopic Score, Partial Mayo Score, ulcerative colitis

## Abstract

**Background:**

The number of elderly patients with ulcerative colitis (UC) has been increasing worldwide. Complications are common in elderly patients who undergo colonoscopy, raising doubts about whether colonoscopy should be performed in the same way in this age group as in younger patients. The aim of this study was to determine the safety of full bowel preparation and colonoscopy in elderly patients with UC.

**Methods:**

We retrospectively reviewed a cohort of patients with UC who had visited any of the 12 hospitals and were registered in our inflammatory bowel disease database. We compared complications associated with colonoscopy and bowel preparation and relapse of UC after colonoscopy in 133 patients aged ≥65 years with UC (the elderly group) and 116 randomly selected patients aged <65 years with UC (the non‐elderly group).

**Results:**

Nine elderly patients were not referred for colonoscopy by their physicians because of poor performance status or advanced age. There was no significant between‐group difference in the complication rate (*p* = 0.57) or frequency of relapse of UC after colonoscopy (*p* = 0.67).

**Conclusions:**

The findings of this study indicate that colonoscopy can be performed as safely in elderly patients with UC as in their younger counterparts. However, our results also indicate that colonoscopy is often avoided in elderly patients, possibly because of concerns about safety.

## INTRODUCTION

Ulcerative colitis (UC) is a chronic inflammatory disease arising from an interaction between genetic and environmental factors, and its incidence is rising worldwide.^1^ ^.^ ^2^ Along with the increasing total number of patients with UC, the number of elderly patients with the disease is continuing to increase.^3^ There are differences in the disease course, treatment efficacy, possible side effects of therapy, and not least, the extent to which quality of life is affected between elderly patients with inflammatory bowel disease (IBD) and their younger counterparts.^4^ These differences suggest that the management of UC may vary according to age group.

Accurate assessment of the extent of the disease and its activity is crucial for monitoring the clinical course and optimization of treatment in patients with UC.^5^ Monitoring of disease activity should be based on a combination of clinical symptoms and findings of laboratory investigations, colonoscopy, and imaging examinations.^1^ Colonoscopy remains the reference standard for assessment of the activity and extent of UC, which involves both the colonic and rectal mucosa.^5^ There is extensive evidence showing the importance of both mucosal healing and histological healing and their excellent correlation with reduced risk of relapse^6^ and hospitalization.^7^ However, colonoscopy is an invasive procedure that may cause discomfort and increase the risk of bowel perforation, and repeated colonoscopy procedures are unpopular with patients.^8^ Elderly patients are likely to have declining immune function and metabolic capacity and to be at increased risk of complications after colonoscopy. Therefore, there is some doubt as to whether they should be managed in the same way as younger patients. Little is known about the effect of age on the risks associated with colonoscopy in patients with IBD.^9^ The aim of this study was to determine the safety of full bowel preparation and colonoscopy in elderly patients with UC. The indications for endoscopy in elderly patients with UC are discussed.

## MATERIALS AND METHODS

This retrospective study included a cohort of patients with IBD who visited any of the 12 hospitals in the Gunma Prefecture in Japan and are registered in our IBD database　between January 1, 2021, and December 31, 2021. In all patients, UC was diagnosed using established endoscopic, histological, and clinical criteria, and its severity was defined according to the criteria set down by the Research Committee on Inflammatory Bowel Disease in Japan.^10^ In the absence of an agreed definition, we defined “elderly” as age ≥65 years, which is consistent with previous reports.^11^, ^12^, ^13^ Using this definition, we reviewed the medical records of elderly and non‐elderly patients who were registered in the Gunma Prefecture IBD database between January 1, 2021, and December 31, 2021. Patients with UC aged 20–64 years were identified using the minimization method described by the RAND function in Excel and enrolled as a non‐elderly group. The following exclusion criteria were applied: previous colonic resection; follow‐up duration of less than 6 months; suspected cytomegalovirus infection in the colon or other type of infectious colitis; only the first colonoscopy was included patients who underwent multiple colonoscopies during the study period; incomplete colonoscopy because of poor bowel preparation; and patient's request for discontinuation.

The study was conducted in accordance with the amended Declaration of Helsinki and approved by the Gunma University Hospital Institutional Review Board. Written informed consent was obtained from all patients before the colonoscopy was performed.

The trial is registered in the UMIN Clinical Trial Registry as UMIN 000048092.

### Bowel preparation and colonoscopy procedure

All patients were required to ingest polyethylene glycol solution (e.g., Moviprep, Nifrec, Muben, and Magcolor) on the morning of the procedure. In an effort to ensure adequate bowel preparation, stool color was assessed before each colonoscopy using a score sheet, and additional polyethylene glycol solution was used if deemed necessary by the nurse.

No details on the types of colonoscope used were available; however, operators tended to prefer the use of a pediatric colonoscope. All the procedures were performed by or under the supervision of an experienced gastroenterologist who had performed at least 100 colonoscopy procedures.

### Safety endpoints

The safety endpoints were as follows: incidence, severity, and outcomes of complications of colonoscopy, such as bleeding, perforation, and laceration; complications associated with bowel preparation, such as vomiting and intestinal obstruction; and the rate of relapse of UC after colonoscopy. We often encounter patients with UC who complain of worsening symptoms after colonoscopy; whether these complaints reflect the burden of bowel preparation or the colonoscopy procedure itself is unclear.^14^, ^15^ We assessed the endoscopic severity, clinical severity, biopsy findings, and bowel preparation method used in patients who developed endoscopic complications or had a relapse of UC after colonoscopy. The follow‐up period was set at 1 month from colonoscopy. Finally, we investigated the characteristics and prognosis of patients who were unable to undergo colonoscopy or were deemed unfit for the procedure by the attending physician because of history or conditions from which recovery was not expected. The follow‐up period for such patients was set at one year from colonoscopy.

Endoscopic evaluation of disease inflammatory activity was performed using the Mayo Endoscopic Score (MES)^16^ and Ulcerative Colitis Index of Severity.^17^ Clinical disease activity was evaluated by the Partial Mayo Score (pMayo) and classified as remission (0–1), mild (2–4), moderate (5–6), or severe (7–9).^18^ Relapse was　defined as an increased two or more　points in pMayo. We also assessed the Eastern Cooperative Oncology Group's performance status as translated into Japanese by the Japan Cooperative Oncology Group.^19^

### Statistical analysis

Categorical variables were compared between the groups using Fisher's exact test. And the comparison of continuous variables was used t‐test. All statistical analyses were performed using JMP Pro 14 software (SAS Institute Inc.). A *p*‐value <0.05 was considered statistically significant.

## RESULTS

### Patient characteristics

**FIGURE 1:**
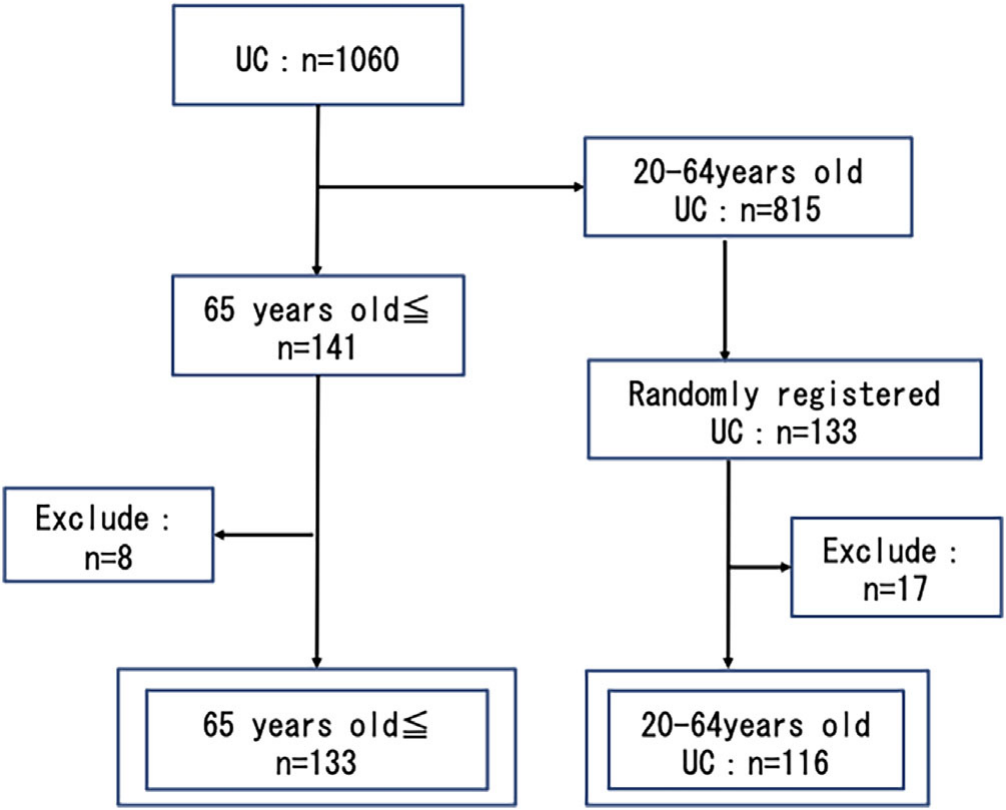
Patient selection algorithm: In total, 1060 patients with ulcerative colitis were identified in the database. Of these, 141 were elderly. Eight elderly patients were not enrolled because of post‐surgery, death, or loss of follow‐up. One hundred and thirty‐three patients who were not elderly were selected at random for inclusion in the study using the RAND function in Excel. Seventeen of the non‐elderly patients were excluded because of post‐surgery or loss of follow‐up.

The database search identified 1060 patients with UC, 141 of whom were elderly; eight of these patients were excluded because they were post‐surgery or were lost to follow‐up, leaving data for 133 patients available for analysis. One hundred and thirty‐one non‐elderly patients were randomly selected for inclusion in the study using the RAND function in Excel; 17 patients were excluded because they were post‐surgery or were lost to follow‐up, leaving data for 116 patients for analysis (Figure 1).

The demographic data are summarized in Table 1. Although there was a significant between‐group difference in age by definition and a significant difference in performance status, there was no significant between‐group difference in any other variable. And, we compared the characteristics between the selected control group and whole non‐elderly patients and show there were no significant differences (data not shown). The selected patients represent the whole non‐elderly UC patients.

**TABLE 1:** Patients’ characteristics.

Items	Elderly UC (*n* = 133)	Non‐elderly UC (*n* = 116)	*p*‐Value
Male	69 (52%)	68 (58%)	ns
Median age (years)	73.4 (65–90)	42 (21–63)	*p* < 0.05
Performance status	0 (0)	0.20 (0–3)	*p* < 0.05
Types of diseases			
Proctitis	25 (19%)	25 (22%)	ns
Left‐sided colitis	39 (29%)	30 (26%)	ns
Total colitis	69 (52%)	61 (53%)	ns
Mayo Endoscopic Score			
0	87 (65%)	65 (56%)	ns
1	19 (11%)	23 (20%)	ns
2	20 (11%)	19 (16%)	ns
3	7 (5%)	4 (3%)	ns
UCEIS			
0‐1	104 (78)	87 (75)	ns
2‐4	22 (17)	25 (22)	ns
5‐6	5 (4)	3 (3)	ns
7‐8	2 (2)	1 (1)	ns
Partial Mayo score			
0–1 (Remission)	99 (73%)	77 (67%)	ns
2–4 (Mild)	6 (5%)	13 (11%)	ns
5–6 (Moderate)	13 (10%)	10 (9%)	ns
7–9 (Severe)	5 (4%)	7 (6%)	ns
Medication at the moment of colonoscopy			
5‐aminosalicylates agents	123 (91%)	96 (83%)	ns
Steroids	6 (5%)	4(3%)	ns
Immunomodulators	23 (17%)	16 (14%)	ns
Anti‐tumor necrosis factor agents	16 (12%)	22 (19%)	ns
Ustekinumabu	0 (0%)	1 (1%)	ns
Vedolizumab	2 (2%)	1 (1%)	ns
Tofacitinib	2 (2%)	2 (2%)	ns
Laboratory data			
C‐reactive protein (mg/dl)	0.22 (0.01–3.1)	0.19 (0.01–4.91)	ns
Alb (g/dl)	4.1 (3.1–5.0)	4.69 (2.9–5.2)	ns

The most common presentation of the disease was total colitis, followed by left‐sided colitis, and the least common was proctitis. Clinical remission was common in both groups, and the Mayo Endoscopic Score was similar in the two groups.

Pharmacological treatments documented at the time of colonoscopy were oral and/or topical 5‐aminosalicylates, oral and/or topical steroids, immunomodulatory agents, and anti‐tumor necrosis factor agents, which were used, respectively, in 91%, 3%, 17%, and 12% of the elderly patients and in 83%, 3%, 14%, and 19% of the non‐elderly patients.

### Safety indicators

**FIGURE 2:**
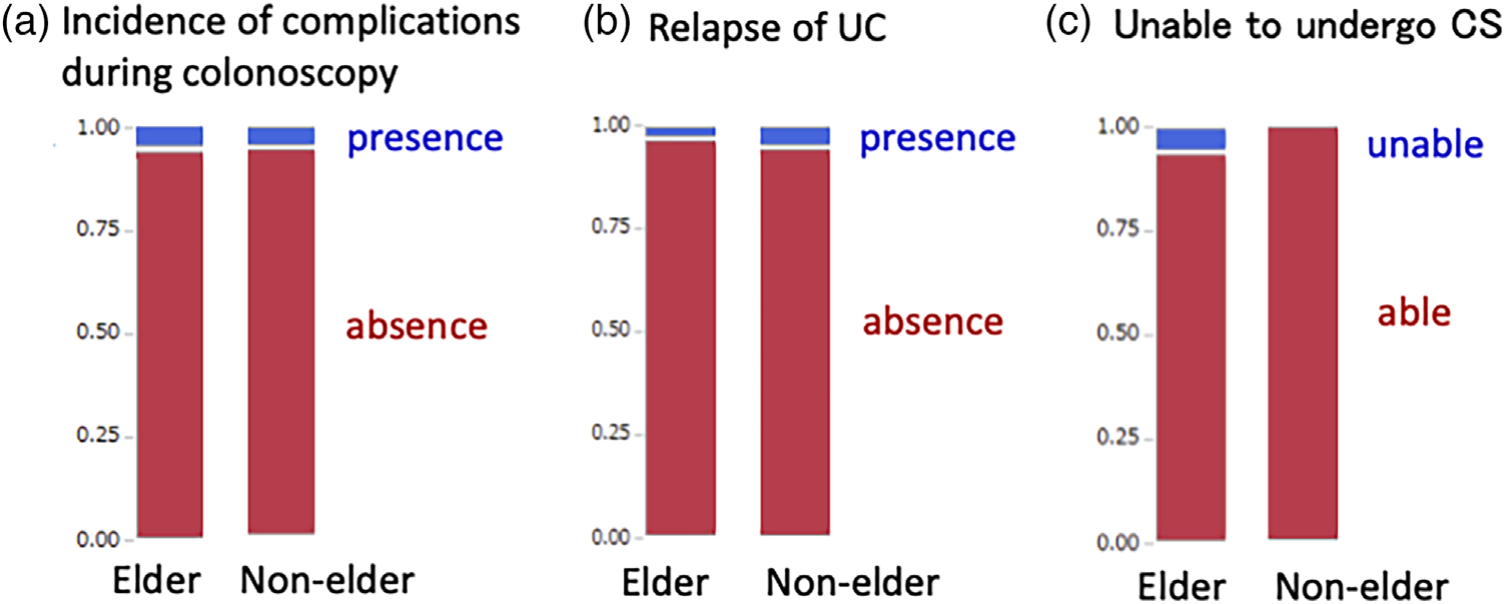
Indicators of safety: (a) Incidence of complications during colonoscopy. There was no significant correlation between the elderly group and the non‐elderly group (*p* = 0.57). (b) Relapse of ulcerative colitis. There was no significant correlation between the elderly group and the non‐elderly group (*p* = 0.67). (c) Unable to undergo colonoscopy. There was a significant correlation between the elderly group and the non‐elderly group (*p* = 0.016).

The safety indicators are shown in Table 2 and Figure 2. Four elderly patients and four non‐elderly patients had complications during colonoscopy. Vomiting was the most common complication, with one case each of bleeding at a biopsy site and abdominal pain. None of the complications required hospitalization. There was no significant correlation between complications in the elderly group and the non‐elderly group (*p* = 0.57).

**TABLE 2:** Indicators of safety.

Incidence of complications during colonoscopy	Age	Mayo score	Treatment	Reason
Elder patient ①	78	0	5‐ASA	Vomiting
Elder patient ②	72	0	5‐ASA	Vomiting
Elder patient ③	71	2	5‐ASA, IM	Vomiting
Elder patient ④	90	0	5‐ASA	Abdominal pain
Non‐elder patient ①	48	2	5‐ASA, IM	Biopsy bleeding
Non‐elder patient ②	53	0	5‐ASA	Vomiting
Non‐elder patient ③	42	0	5‐ASA	Vomiting
Non‐elder patient ④	39	1	5‐ASA	Vomiting

Relapse of UC	Age	Mayo score	Treatment	Progress
Elder patient ①	76	5	5‐ASA, PSL	Moderate→severe
Elder patient ②	85	5	5‐ASA	Hemorrhagic shock
Non‐elder patient ①	21	6	5‐ASA, IM	Moderate→severe
Non‐elder patient ②	23	5	5‐ASA, TNF	Moderate→severe

Unable to undergo colonoscopy	Age	Mayo score	Treatment	Reason
Elder patient ①	83	0	5‐ASA	Dementia
Elder patient ②	86	0	5‐ASA	AAA postoperative
Elder patient ③	71	0	5‐ASA	After infarction
Elder patient ④	89	0	5‐ASA	At his own request due to age
Elder patient ⑤	78	0	5‐ASA	Dementia
Elder patient ⑥	73	0	5‐ASA	After stroke
Elder patient ⑦	80	0	5‐ASA	At his own request due to age
Elder patient ⑧	80	1	5‐ASA	Parkinson disease
Elder patient ⑨	73	0	Untreated	At his own request due to age

Two elderly patients and two non‐elderly patients had a relapse of moderate or severe UC after colonoscopy. One of these patients developed hemorrhagic shock. Treatment at the time of colonoscopy included steroids, immunomodulator, and anti‐tumor necrosis factor therapy. There was no significant correlation between the disease relapse rate in the elderly group and that in the non‐elderly group (*p* = 0.67).

### Risk factors for colonoscopy‐related complications in elderly patients

Nine elderly patients were deemed by their physicians not to be candidates for colonoscopy because of a history of stroke or dementia. Remission was achieved by treatment with a 5‐aminosalicylate in these patients. No patients in the non‐elderly group were deemed by their physicians not to be candidates for colonoscopy.

Risk factors for colonoscopy complications in elder patients are shown in Table 3. The MES and pMayo were 1.8 and 2.0, respectively, in the group with complications and 0.7 and 0.3 in the group without complications; the between‐group differences in scores were statistically significant. A univariable logistic regression analysis was performed (odds ratio 17.65, *p* = 0.02 and odds ratio 18.89, *p* = 0.02, respectively).

**TABLE 3:** Risk factors for colonoscopy complications in elder patients.

	Complication (*n* = 6)	Non‐complication (*n* = 127)
Biopsy	4 (67%)	49(39%)
Mayo endoscopic score	1.8 (0–3)	0.7 (0–3)
Partial mayo score	2.0 (0–8)	0.3 (0–7)
Laxative	6 (100%)	124 (98%)
Performance status	0.83 (0–2)	0.17 (0–3)
Medication at the moment of colonoscopy		
5‐aminosalicylates agents	6 (100%)	116 (91%)
Steroids	1 (17%)	5 (4%)
Immunomodulators	2 (33%)	21 (17%)
Biology	2 (33%)	18 (14%)
Laboratory data		
C‐reactive protein (mg/dl)	0.43 (0.02–1.5)	0.22 (0.01–4.91)
Alb (g/dl)	3.7 (3.0–4.1)	4.1 (2.9–5.0)

There were no significant between‐group differences in the biopsy rate, content of bowel preparation, medicine of UC, or results of laboratory investigations. However, the group with complications tended to have a higher biopsy rate and a higher C‐reactive protein level and were more likely to be on immunosuppressive treatment.

### Patients who were unable to undergo colonoscopy　

Patients deemed not to be candidates for colonoscopy were a significantly correlation between the elderly group and the non‐elderly group (*p* = 0.016). Nine patients were deemed by their physicians to be ineligible for colonoscopy because of a poor performance status or a history of stroke or dementia (patients in whom colonoscopy could not be completed because of severe pain or other reasons are not included in this group). These patients were followed for one year after enrollment in this study. One patient had a relapse of UC and underwent proctocolectomy. The remaining eight patients are progressing without problems, including no relapse of UC.

## DISCUSSION

Although there have been a few reports on colonoscopy in elderly patients with UC,^20^, ^21^, ^22^ most are related to surveillance. It has been reported that routine endoscopy undergone in very elderly patients (aged 85 or older) were at higher risk of adverse events than those aged less than 85.^23^ This study is limited to elderly and young patients with ulcerative colitis. Therefore, results may differ from studies that include healthy subjects. Also, the age of the elderly is defined as 65 years and older (WHO's definition of elderly), so that would also make a difference. As noted in the discussion, it should be recognized that there are many elderly people who are well past the age of 65. This study shows that being an elderly patient (over 65) does not increase the risk of colonoscopy.

Complications during colonoscopy were mostly minor, with no cases requiring hospitalization.　All cases of disease relapse occurred in patients with moderate disease. Therefore, it is unclear whether the relapse of UC was an adverse effect of colonoscopy or if it was part of the natural course of the disease. Either way, the findings of this study show that colonoscopy can be performed as safely in elderly patients with UC as in their younger counterparts. However, our results also suggest that colonoscopy is avoided by a significant proportion of elderly patients and that the safety of the procedure might be a consideration in this aged group. It is not possible to make recommendations regarding contraindications to colonoscopy in elderly patients based on our present findings alone. It seems to be insufficient to evaluate all the patients whom the physician considered to be inappropriate for colonoscopy, because there might be no information or description in the medical record about the process of avoidance of colonoscopy in some cases, or colonoscopy was not considered at all in case of high risk. In Japan, the severity of UC is reported to be significantly greater in patients who are older at the time of disease onset,^4^, ^12^ and UC‐related mortality is also reported to be higher in these patients. Indeed, one of the patients in our study who avoided colonoscopy experienced a severe relapse and underwent surgery. Furthermore, the risk of UC‐associated colorectal cancer has been shown to increase with the duration of UC.^24^, ^25^, ^26^ Therefore, the importance of surveillance colonoscopy increases with age. The challenge for the future is to identify the characteristics of elderly patients with UC that may warrant avoidance of colonoscopy. The American Society for Gastrointestinal Endoscopy guidelines state that colonoscopy for colorectal cancer screening is not necessary in patients over 85 years of age.^27^ Thus, surveillance colonoscopy in patients with UC may not need to be based on age. However, there is a need for a clear statement concerning this issue in the IBD‐related guidelines.

This study has some limitations. First, it had a retrospective design, and although bowel preparation methods were mostly standardized, the products used were not. Furthermore, mild worsening of symptoms may not have been noted in the medical records. Second, the risk of complications of colonoscopy may depend on the skills of the operator and the type of endoscope used; these factors could not be matched because the study included data from multiple centers. The use of anti‐coagulant medication and sedation also might be possible confounders. This study did not evaluate the use of those. However, we believe our findings are important because they were obtained in a real‐world setting. An important limitation is that the indications for colonoscopy probably differ between elderly and non‐elderly patients. Doctors would not choose to perform colonoscopies more often in elderly patients with severe diseases. The risk is likely to increase as the frequency of colonoscopies increases. The inclusion of only a small number of patients with severe symptoms is also a limitation of this study. Finally, it needs to be acknowledged that there are many people in the community who are over the age of 65 years, remain healthy, and cannot be considered elderly.

## CONCLUSIONS

We have demonstrated that colonoscopy can be performed as safely in elderly patients with UC as in non‐elderly patients. In the near future, we will be performing a prospective observational study to determine the conditions that would warrant colonoscopy in elderly patients with UC, taking into account their general state of health, and the severity of UC.

## CONFLICT OF INTEREST STATEMENT

Toshio Uraoka is a Deputy Editor‐in‐Chief of DEN Open. The rest of the authors declare no conflict of interest.
